# Discrete Repetition Effects for Visual Words Compared to Faces and Animals, but No Modulation by Expectation: An Event‐Related Potential Study

**DOI:** 10.1111/ejn.70047

**Published:** 2025-03-03

**Authors:** Bingbing Song, Werner Sommer, Urs Maurer

**Affiliations:** ^1^ Institute for Brain Research and Rehabilitation South China Normal University Guangzhou China; ^2^ Department of Psychology The Chinese University of Hong Kong Hong Kong China; ^3^ Department of Psychology Humboldt‐Universität zu Berlin Berlin Germany; ^4^ Faculty of Education National University of Malaysia Kuala Lumpur Malaysia; ^5^ Life Science Imaging Center Baptist University Hongkong Hong Kong China; ^6^ Brain and Mind Institute The Chinese University of Hong Kong Hong Kong China

**Keywords:** expectation, predictive coding, repetition suppression, visual expertise

## Abstract

Repetition suppression (RS) refers to the reduction of neuronal responses to repeated stimuli as compared to nonrepeated stimuli. The predictive coding account of RS proposes that its magnitude is modulated by repetition probability (P(rep)) and that this modulation increases with prior experience with the stimulus category. To test these proposals, we examined RS and its modulation by P(rep) for three stimulus categories for which participants had different expertise (Asian faces, written Chinese words and animals) using EEG. Cantonese speakers watched paired stimuli (S1–S2) of a given category with S2 being the same or a different stimulus as S1. Attributes of S1 (e.g., the sex of the first face) served as a cue for the repetition probability of S2. There were significant repetition effects and distinct topographic distributions across stimulus categories. Repetition effects in the N250 component were present in all stimulus categories, but in words, they appeared earlier and showed distinct topographic patterns compared to faces and animals. These results suggest that repetition effects differ between stimulus categories, presumably depending on prior experience and stimulus properties, such as spatial frequency. Importantly, we failed to find evidence for effects of P(rep) across any of the three categories. These null findings of P(rep) effects are putatively indicating an absence of expectancy modulation of repetition effects.

## Graphical abstract

Visual words, faces and animals showed consistent N250r effects with distinct topographic distributions. The repetition effects for words occurred earlier than for faces and animals. We failed to find EEG evidence for repetition effects modulated by expectation in any of the three visual stimulus categories.

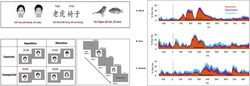

**Abbreviations**
Exp_Alt: expected alternations
Exp_Rep: expected repetitions
GDS: global duration statistics
GFP: global field power
ICA: independent component analysis
P(rep): repetition probability effect
PC: predictive coding theory
RS: repetition suppression
S1/S2: the first/second stimulus
TANOVA: topographic analyses of variance
Unexp_Alt: unexpected alternations
Unexp_Rep: unexpected repetitions

## 1 Introduction

How sensory information is processed in the neurocognitive system depends not only on the current sensory input but also on the previous experiences of the individual. A well‐known phenomenon taken as evidence for the statement above is the diminished neural response to repeated stimuli compared to nonrepeated stimuli, known as repetition suppression (RS). RS has been consistently reported across a wide range of neural signals, sensory modalities, stimulus properties and time scales (Feuerriegel 2024; Grill‐Spector et al. 2006; Grotheer and Kovács 2016; Vogels 2016).

### 1.1 Electrophysiological Studies of Repetition Effects

EEG‐derived event‐related potentials (ERP) components like the N170, N250r and N400 have been informative in investigating the temporal characteristics of repetition effects. The N170 component, elicited by faces and words, peaks around 170 ms and shows more consistent repetition effects for written words and letter strings than for individual faces (e.g., Cao et al. 2015; Maurer et al. 2023; Rugg and Nagy 1987). The N250r, an increased negativity for repeated as compared to unrepeated faces around 200–300 ms, has been found to be larger for familiar than for unfamiliar faces (see review Schweinberger and Neumann 2016). The N250r effect is not specific to faces but can also be elicited by common objects and familiar buildings (Gruber and Müller 2005; Engst et al. 2006). The effects of repetition on the N250 for written words are inconsistent. Whereas several studies reported a reduction in N250 amplitude for repeated words (Holcomb and Grainger 2006; Kiyonaga et al. 2007; Huang et al. 2022; Maurer et al. 2023), others, such as Pfütze et al. (2002) and Pickering and Schweinberger (2003), reported an increased N250 amplitude for repeated words of familiar written names. This inconsistency might be due to several factors, including the different electrodes and reference used across studies (Maurer et al. 2023) and different timings of the effects. The N400, linked to semantic processing, is usually attenuated for repeated words compared to nonrepeated words (Bentin et al. 1985; Van Petten et al. 1991; Rugg 1985, 1990).

Previous studies have shown the influence of familiarity on repetition effects across various visual stimuli, such as faces, buildings and words (e.g., Engst et al. 2006; Nie et al. 2016; Maurer et al. 2023; Guillaume et al. 2009). Engst et al. (2006) demonstrated differential repetition effects between faces and buildings: while both categories elicited topographically similar N250r components, buildings showed smaller amplitudes than faces. The N400 responses revealed category‐specific scalp topographies, indicating distinct neural mechanisms for face versus building recognition. Guillaume et al. (2009) demonstrated a distinction between repetition effects for familiar faces and object drawings. Both faces and objects showed a diminished N170 when primed, while later repetition effects at 400–600 ms at central and frontal sites were predominantly left‐sided for objects and right‐sided for faces. More recently, a recent EEG study by Maurer et al. (2023) showed a reduced negativity in the falling flank of the N1 after repeated compared to unrepeated visual words. Such repetition‐related N1 reduction was more pronounced for familiar Chinese than unfamiliar Korean words. However, most previous studies examining repetition effects varied in stimulus categories used, settings of the tasks (e.g., duration of repetition) or procedural specifics (Leuthold and Sommer 1993). The extent to which the repetition effects are modulated by stimulus familiarity remains unclear unless a comparable paradigm is used. Therefore, by using a uniform experimental within‐subject design, the first aim of the present study was to explore the influence of stimulus familiarity on repetition effects.

### 1.2 The Predictive Coding (PC) Account of RS

Despite its widespread use, the neurocognitive mechanisms reflected in RS remain controversial (Epstein and Morgan 2012). Early theories proposed that RS was a result of bottom‐up processes, such as neural fatigue (Grill‐Spector and Malach 2001; Miller and Desimone 1994), sharpening of neural representations (Desimone 1996; Wiggs and Martin 1998) or facilitation of neural processing (Henson and Rugg 2003; James and Gauthier 2006). These theories suggest that the repetition of a stimulus leads to a reduced neural response due to the adaptation of neural circuits to that stimulus. However, recent theories have emphasized the role of top‐down processes in RS (Auksztulewicz and Friston 2016; Summerfield et al. 2008). According to the PC theory, the brain constantly generates predictions about incoming sensory information based on prior experience and aims to minimize discrepancies (prediction errors) between actual and predicted sensory states (Clark 2013; Friston 2005; Rao and Ballard 1999). The PC model can account for a wide range of perceptual and neural phenomena, such as RS, especially when examining the role of expectation in perception (for reviews, see Feuerriegel et al. 2021; Walsh et al. 2020). From this perspective, RS may represent a reduction of prediction error signals due to the fulfilment of perceptual expectations that are biassed towards recently encountered stimuli (Auksztulewicz and Friston 2016; Summerfield et al. 2008).

The modulation of RS by top‐down expectations constitutes a critical hypothesis of PC (Walsh et al. 2020). Indeed, emerging empirical evidence supports the PC account. An early fMRI study by Summerfield et al. (2008) introduced a standard block repetition probability paradigm, presenting pairs of faces sequentially that could either be the same (repeat) or different (nonrepeat); these trials were presented within separate blocks in which most of the trials were either repeats (75%) or—alternatively—nonrepeats (75%). The magnitude of RS (repeat vs. nonrepeat) in the fusiform face area (FFA) was greater in blocks of trials when repetitions were frequent rather than infrequent, indicating that RS is increased by expectation. Summerfield et al. argued that the modulation of RS by expectation reflects the neural consequence of perceptual predictive processing: As participants have a higher expectation for encountering a stimulus repetition in repetition blocks, this leads to a stronger RS magnitude compared to alternation blocks where stimulus repetitions are rare and (relatively) surprising (although see Larsson and Smith 2012). Thus, the P(rep) effect may provide a window into understanding the influence of prior expectations on sensory processing.

The P(rep) effect has been replicated with various neuroimaging methods for faces (Amado and Kovács 2016; Kovács et al. 2013; Larsson and Smith 2012; Summerfield et al. 2008), familiar roman letters (Grotheer and Kovács 2014), familiar scripts (Li and Kovács 2022), common visual objects (Mayrhauser et al. 2014), human voices (Andics et al. 2013) and stress of spoken words (Honbolygó et al. 2020). In contrast, the P(rep) effect has not been replicated in several other studies (e.g., Kaliukhovich and Vogels 2011; Kovács et al. 2013; Grotheer and Kovács 2014; Li and Kovács 2022). The discrepant effects of P(rep) may be attributable to several experimental variables, such as differences in stimulus types, task designs and repetition intervals. Among these factors, differences in the stimuli used may play a crucial role. The P(rep) effects appear to be present for certain stimuli like faces and letters (Kovács et al. 2013; Grotheer and Kovács 2014; Li and Kovács 2022) but not for common objects, unfamiliar false fonts or novel script (Kaliukhovich and Vogels 2011; Kovács et al. 2013; Mayrhauser et al. 2014; Grotheer and Kovács 2014; Li and Kovács 2022). A possible explanation for this inconsistency has been proposed by Grotheer and Kovács (2014), namely, that P(rep) effects depend on prior experience with the stimulus category. Increased prior experience with a stimulus category may strengthen associations between perceptual features and increase P(rep) effects. However, direct comparisons of P(rep) effects across stimulus categories that differ in visual expertise using comparable experimental paradigms are lacking. Such findings could offer direct evidence of whether predictive processing is restricted to specific types of stimuli, like visual words, or whether predictive processing is more generally dependent on prior experience with the stimuli.

Importantly, while the P(rep) effect has been found in several fMRI experiments (e.g., Grotheer and Kovács 2014; Li and Kovács 2022), repetition and expectation may influence distinct perceptual and cognitive processing stages. The sluggishness of the BOLD response in fMRI makes it difficult to determine the exact timing or functional localization of such effects (Glover 2011). Previous fMRI studies reporting P(rep) effects may contain several repetition and expectation effects across time points (see review, Feuerriegel et al. 2021). In contrast, ERPs with their high temporal resolution may allow to reveal the temporal dynamics of the repetition and expectation effects. A few EEG/MEG studies have examined the modulation of RS by expectation in visual words (Song et al. 2024; Summerfield et al. 2011), faces and visual gratings (Barbosa and Kouider 2018), as well as pure tones (Todorovic et al. 2011). For instance, a recent EEG study by Song et al. (2024) employed a block RS design as in Summerfield et al. (2008) and found that expectation modulates RS during visual word processing only after 300 ms but not at early stages. Findings of this study align with reports that RS typically starts between 100 and 250 ms poststimulus in the visual modality (Henson et al. 2004; Schendan and Kutas 2003; Schweinberger et al. 2004; Summerfield et al. 2011) and between 40 and 60 ms in the auditory modality (Todorovic and de Lange 2012). In contrast, the modulation of RS by expectation appeared only later, after 300 ms for faces (Summerfield et al. 2011) and between 100 and 200 ms for pure tones (Todorovic and de Lange 2012). These findings support a role of probability‐based top‐down expectation in generating (or at least modulating) RS effects. Other studies reported independent and additive effects of RS and expectation, rather than interactive effects (Feuerriegel et al. 2018; Kaliukhovich and Vogels 2011; Todorovic and de Lange 2012; Vinken et al. 2018). For instance, an ERP study with faces by Feuerriegel et al. (2018) demonstrated that, while the main effects of repetition and P(rep) were identified across several time windows, no interactions between these factors were observed. These findings are consistent with a two‐stage model of response suppression (Grotheer and Kovács 2015), which suggests that face recognition involves two distinct predictive processing stages, low‐level predictions, driven by recent stimulus exposure, and operating within the visual hierarchy up to the FFA, and higher level expectations generated in frontal areas (Amado and Kovács 2016; Choi et al. 2017; Grotheer and Kovács 2015).

It is essential to note that previous studies showed P(rep) effects during block‐wise manipulations of repetition probability, that is, varying expectations. However, in such designs, stimulus repetition and expectation are not independent and may result in confounded and additive effects, where expectation effects may differ across block types (Grotheer and Kovács 2015). Hence, repetition and expectation effects require independent testing by manipulating repetition probability. Event‐related repetition probability designs provide an effective alternative for examining how expectations arise from associations between cues and the repetition probability of upcoming stimuli (Grotheer and Kovács 2015; Todorovic and de Lange 2012). These designs enable the orthogonal manipulation of stimulus repetitions and expectations, allowing to differentiate the effects of RS from those of perceptual expectations.

### 1.3 The Current Study

This study investigated category‐specific modulations of RS effects, with particular emphasis on probability‐dependent repetition effects (P(rep)), using a cued repetition probability paradigm and ERP measures. We employed three distinct stimulus categories—faces, written words and animal images—to examine whether P(rep) effects are category‐selective or contingent upon perceptual expertise. Written words and faces were selected because both categories are visual stimuli in which literate adults have high levels of expertise. Processing of faces is similar to visual words, as shown by an enlarged N170 component compared to control stimuli and by the location of specialized processing in the inferior‐temporal cortex (Davies‐Thompson et al. 2016; Moret‐Tatay et al. 2020). These characteristics make face and visual word stimuli well suited to investigate predictive processing. In contrast, the category of animals (i.e., fish and bird) was chosen because fish and birds are representative objects with which the average individual has a relatively low level of visual expertise (Ćepulić et al. 2018) compared to other common objects such as cars. This selection allowed us to examine P(rep) effects in visual stimuli with low visual expertise. We recorded EEG in a unitary event–related repetition probability paradigm, where the subcategory of the first stimulus (S1) indicated the repetition probability of the second stimulus (S2), assuming that high or low repetition probabilities would increase or decrease the expectation of stimulus repetition. This design allowed to orthogonally assess the effect of stimulus repetitions versus alternations when they were expected or unexpected and—most importantly—whether these factors of repetition and expectation had independent or interactive effects on ERPs. To identify the temporal dynamics of repetition and expectation effects in the ERPs, we utilized a data‐driven data analysis method, that is, point‐by‐point topographic analyses of variance (TANOVA).

We expected to see repetition effects and their modulation by expectation in the repetition probability design for visual word and face recognition. According to the visual expertise hypothesis, such repetition probability effects should be diminished or absent in pictures of animals, for which prior experience should be less pronounced than for faces and words. In addition, we expected to see distinct repetition effects (in terms of timing and scalp distribution) for written words, faces and animals, since familiarity has been shown to modulate repetition effects for faces and visual words (e.g., Nie et al. 2016; Maurer et al. 2023).

## 2 Methods

### 2.1 Participants

We report data from 33 right‐handed, native Chinese (Cantonese) speakers (21 females, mean age = 19.39 years, range = 18–23 years). Data from three additional participants were excluded from the analysis due to poor data quality (the number of remaining trials was below 50% of all trials). All participants reported regular reading ability and normal or corrected‐to‐normal visual acuity. Prior to the experiment, all participants were informed about the experimental procedures and provided written informed consent. The research protocol was approved by the Joint Chinese University of Hong Kong‐New Territories East Cluster Clinical Research Ethics Committee. Sample size was initially determined based on previous RS studies showing large effect sizes (Maurer et al. 2023). We then conducted a power analysis in PANGEA (Westfall 2015) for a 2 × 2 repeated‐measures analysis of variance (ANOVA) design to detect the expected effect size (Cohen's *d* = 0.62) of interaction effects between repetition and expectation in ERP amplitudes, aiming for a power level of 0.78 (α = 0.05, two‐tailed; *N* = 36). This expected effect size estimate aligns with our recent findings showing expectation‐by‐repetition interaction effects with Cohen's *d*‐values up to 0.59 in a similar paradigm (Song et al. 2024).

### 2.2 Stimuli

**FIGURE 1:**
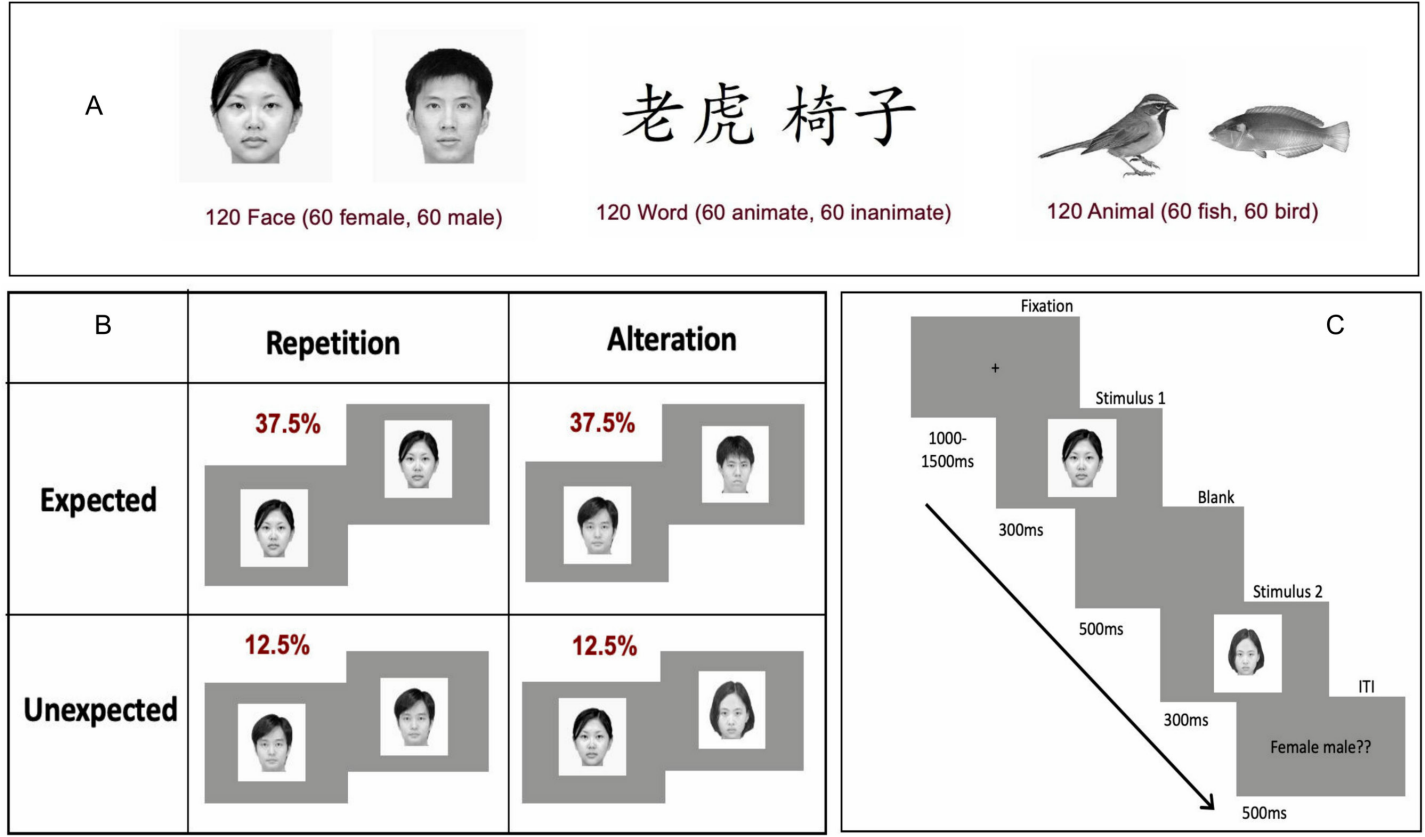
Examples for stimulus types (A), experimental design and conditions (B), and trial sequence (C). In the illustrated case, a female S1 face indicates a high probability of repetition (75%) and a low probability of alternations (25%). At the end of the trial, S1 has to be categorized as male or female: 37.5% trials are expected to either repeat or alternate, while 12.5% indicates the proportion of trials that repeat or alternate unexpectedly.

A pilot study preceded the main experiment to establish equivalent discriminability across stimulus categories.^1^ Based on these pilot data, we selected 120 exemplars per category that had been categorized with 100% accuracy as male/female for faces, animate/inanimate for words and bird/fish for animals. This stimulus selection procedure ensured comparable task difficulty and discriminability across all three categories. Therefore, a total of 360 stimuli were used in the formal study, including 120 Asian faces (subcategories: 60 males, 60 females), 120 written high‐frequency two‐character Chinese words (subcategories: 60 animate, and 60 inanimate) and 120 animal pictures (subcategories: 60 fish, 60 birds) (for examples, please see Figure 1). The faces were taken from the CUHK Face Sketch Database (CUFS, X. Wang and Tang 2009), while two‐character Chinese words were selected from the SUBTLEX‐CH database (Lai and Winterstein 2020). The mean stroke number of Chinese words was 21.28 (SD = 5.22), and the mean word frequency was 9.09/million (SD = 10.25, range = 1 to 38/million), and character repetition times^2^ in the two‐character word were 4.0 (SD = 2.01). The word frequencies, total stroke numbers and repetition times were matched between animate and inanimate words (Figure S1). The bird and fish images were obtained from the NABirds dataset (Van Horn et al. 2015) and the Fish Image Set (Anantharajah et al. 2014), respectively. All images were cropped, resized, using Adobe Photoshop software, and displayed at the centre of the screen on a uniform white background. The mean luminance values were equated across images using the SHINE toolbox (Willenbockel et al. 2010). At a viewing distance of 70 cm, all stimuli subtended visual angles of approximately 2.45°–2.86°.

### 2.3 Task and Procedure

The experiment was conducted in a sound‐attenuated, darkened room, where participants were seated comfortably and viewed a 144‐Hz computer monitor. We employed a similar repetition probability paradigm as Grotheer and Kovács (2015), incorporating Chinese words, faces and animal images as stimuli. In a repetition probability paradigm, we orthogonally manipulated stimulus repetitions versus alternations and the probability of repetitions versus alternations (expectation). Paired stimuli (pairs of faces, words or animals) consisted of either the same stimulus (repetition) or different stimuli (alternation) but always from the same category. Within each category, subcategories of S1 signalled high or low repetition probabilities (75% vs. 25%). For instance, in the face condition, female S1 faces were associated with a 75% probability of repetition and a 25% probability of alteration. Conversely, male S1 faces indicated a 75% probability of alteration and a 25% probability of repetition as S2. This design should induce participants to form expectations about the likelihood of S2 repetitions or alternations upon identifying S1. As a result, there were four distinct conditions for each stimulus type: expected repetitions, expected alternations, unexpected repetitions and unexpected alternations. The experimental designs for the face, word and animal conditions were identical; the subcategories of visual word (animate or inanimate) and animal (fish or bird) signalled the 75% or 25% repetition probabilities of S2. The categorization task for S1 varied across three conditions: determining the sex of a face (male or female), the animacy of a word (animate or inanimate) and the type of animals (fish or bird). Please note that the S1 category merely signalled the likelihood of the repetition of the S1 stimulus as S2—the probability of the S1 category was always 50%.

Each trial started with a fixation cross, randomized in duration between 1000 and 1500 ms, followed by the sequential presentation of S1 and S2 of a stimulus pair, for 300 ms each and separated by an interstimulus interval of 500 ms, after which the intertrial interval of 500 ms commenced (see Figure 1). Twenty percent of the trials were target trials, which were marked by an additional response slide after the trial. In target trials, participants had to categorize S1 (e.g., female vs. male faces, animate vs. inanimate words, fish vs. bird pictures). Nontarget trials that did not require a categorization response comprised the remaining 80% of all trials. Importantly, both types of trials included conditions of expectation (expected vs. unexpected) and repetition (repetition vs. alternation). Both target and nontarget trials were included in all conditions of the 2 repetition (repetition vs. alternation) × 2 expectation (expected vs. unexpected) design. For each of the three categories (visual words, faces, animals), the distribution was as follows: 180 expected repetition trials, 180 expected alternation trials, 60 unexpected repetition trials and 60 unexpected alternation trials. Prior to the experiment, participants were informed about the relative repetition/alternation probabilities. To minimize adaptation to low‐level features, in each trial, the size of either S1 or S2 (chosen randomly and equiprobably) was reduced by 18%. Trial sequences within each block were presented in randomized orders. Before the experiment, participants completed 16 practice trials, with trial sequences presented in pseudorandomized order.

In a total of 1440 trials, 480 trials were presented in homogeneous blocks for each stimulus type. Each block lasted approximately 25 min, resulting in a total duration of the experiment of 1.5 h. We counterbalanced the order of the assignments of stimulus category and repetition probability across participants.

### 2.4 Data Collection and Analysis

#### 2.4.1 EEG Recordings

The EEG signal was recorded with 64 Ag/AgCl electrodes mounted in a cap at standard 10–10 positions and referenced against CPz. Two horizontal electrooculogram (EOG) electrodes were placed on the outer canthi of the left and right eyes, and one was placed on the infraorbital ridge of the left eye to measure the vertical EOG. All impedances were kept below 20 kΩ. The signals were amplified by an ANT amplifier system (Advanced Neuro Technology, Enschede, Netherlands) with a bandpass of 0.1–70 Hz and a sampling rate of 1000 Hz.

#### 2.4.2 EEG Preprocessing

EEG data were preprocessed using Brain Vision Analyzer software. A bandpass filter of 0.3–30 Hz was applied (fourth‐order zero‐phase shift Butterworth). Bad channels were mainly identified through visual inspection. This involved scanning through the raw EEG data to visually detect any electrodes exhibiting abnormal signals such as excessive noise, flat‐line signals or significant deviations from neighbouring channels. The vertical and horizontal eye movement artefacts were corrected using infomax independent component analysis (ICA), one of the most common methods applied to EEG data. We identified artefactual ICA components using the topographies of blink and lateral eye movement artefacts (polarity reversal). Notably, our analyses focused on the EEG responses to the second stimulus (S2). To this end, the continuous EEG was recalculated to an average reference and segmented into epochs from 150 ms before to 850 ms after the onset of S2. Trials with amplitudes exceeding ±80 μV in any channel were automatically rejected from further analysis. Only nontarget trials were included in the formal analysis. Following baseline correction (−150 to 0 ms), average ERPs were calculated for all conditions (for mean trial numbers per condition see Table 1).

**TABLE 1:** The mean number of remaining trials in the 12 experimental conditions (SDs in parentheses).

Stimulus type	Exp_Rep	Exp_Alt	Unexp_Rep	Unexp_Alt
Written words	130.76 (14.98)	128.82 (17.55)	44.36 (5.49)	43.73 (3.96)
Faces	132.18 (14.89)	131.12 (14.94)	43.88 (4.77)	43.94 (5.15)
Objects	132.70 (13.63)	132.76 (23.61)	44.12 (4.14)	45.3 (4.20)

Abbreviations: Exp_Alt = expected alternations; Exp_Rep = expected repetitions; Unexp_Alt = unexpected alternations; Unexp_Rep = unexpected repetitions.

#### 2.4.3 Data Analysis

ERP data were analysed with TANOVA, a data‐driven method enabling the detection of topographic differences in brain activity between experimental conditions. This analysis compares group means of the standard deviation across electrodes of individual mean ERPs using permutation tests at each time point. Specifically, TANOVA calculates a global dissimilarity index (GDI) based on the differences across all electrodes of (non‐normalized) ERP maps of two conditions and estimates corresponding *p* values (Strik et al. 1998). The GDI quantifies the overall topographical dissimilarity between experimental conditions. It is computed as the square root of the sum of squared differences between the voltage at each electrode for different factor levels, normalized by the number of channels (see Murray, Brunet, and Michel 2008, for details). TANOVA has the advantage of using entire ERP maps in the analysis without the need to bias the analysis by a priori electrode selection (Koenig et al. 2011).

To identify the temporal dynamics of repetition and P(rep) effects following stimulus onset, we employed a timepoint‐by‐timepoint TANOVA on non‐normalized scalp maps separately for each stimulus category with factors Repetition (Repeated vs. Alternated) and Expectation (Expected vs. Unexpected) using a 5000 permutation test. It involves conducting 5000 permutations, where individual participants' topographic maps are randomly reassigned to conditions. For each of the 5000 permutations, the GDI is computed, generating an empirical null distribution of GDI values. The original GDI value obtained from the observed data is then compared to this empirical distribution to determine the probability of obtaining a dissimilarity index larger than the one observed, if the null hypothesis were true. Due to the methodological constraints of TANOVA, which permits analysis of only two within‐subject factors simultaneously, we conducted separate analyses for each stimulus category. Each analysis examined the main effects of repetition and expectation, along with their interaction, rather than implementing a three‐factor analysis as was performed with the behavioural data. We then conducted pairwise contrasts of repetition effects across the three stimulus conditions to examine the common and specific repetition effects present across these categories. Finally, as shared repetition effects were observed in the N250 component (N250r) for visual words, faces and animals, we examined the topographic differences of N250r applying pairwise comparisons on the difference waves (repetition minus alternations) within each time window between the stimulus categories. The TANOVA results were corrected for multiple comparisons through global duration statistics (GDS; Koenig et al. 2011). GDS is a nonparametric, permutation‐based technique that calculates the duration of significant time windows (*p* < 0.05). This method compares the duration of significant topographical differences in the observed data to the distribution of significant durations from the 5000 permutations. Only windows where the observed duration exceeds the 95th percentile are considered reliable. In each analysis, all time points between −150 and 850 ms at all 64 scalp electrodes were included. For the TANOVA, partial eta squared was computed in RAGU by using the amount of variance explained in the difference scalp field by the experimental design. The effect size was derived by first computing the mean topographical distribution differences between repetition and alternate conditions across significant time windows, and followed by random permutation testing to obtain the mean and standard deviation of permutation differences. The final effect size was calculated by normalizing the z‐score of the real versus permuted differences by the square root of the product of condition proportions and sample size.

## 3 Results

### 3.1 Behavioural Performance

The mean accuracy in classifying S1 was 95.78% (SD = 8.02) for faces, 97.61% (SD = 3.88) for written words and 96.20% (SD = 5.72) for animals. A Repetition (two levels, Repetition vs. Alternation) × Expectation (two levels, Expected vs. Unexpected) × Stimulus Type (three levels, Face vs. Word vs. Animal) repeated‐measures ANOVA was conducted on the mean accuracy data. No main effect was significant: Stimulus Type (*F* (2, 64) = 2.02, *p* = 0.15, partial *η* ^2^ = 0.02), Repetition (*F* (1, 32) = 1.65, *p* = 0.21, partial *η* ^2^ = 0.004) and Expectation (*F* (1, 32) = 2.60, *p* = 0.11, partial *η* ^2^ = 0.004). Additionally, the two‐way interactions between Repetition and Type and between Repetition and Expectation, and the three‐way interaction were not significant (all *ps* > 0.15).

The mean reaction time (RT) in correctly determining the category of S1 was 699.89 ms (SD = 151.09) for faces, 771.82 ms (SD = 179.60) for written words and 717.82 ms (SD = 156.70) for animals. A three‐way ANOVA on mean RTs showed significant main effects of Stimulus Type, *F* (1, 32) = 3.55, *p* = 0.04, partial *η* ^2^ = 0.03, and Expectation, *F* (1, 32) = 9.86, *p* = 0.01, partial *η* ^2^ = 0.01. However, the main effect of Repetition, the two‐way interaction of Stimulus Type and Repetition and Repetition and Expectation and the three‐way interactions were all non‐significant (all *ps* > 0.20). Figure S2 presents scatter plots of accuracy and RTs for categorizing S1 for visual words, faces and animals.

### 3.2 TANOVA Results

#### 3.2.1 Repetition Effects

**FIGURE 2:**
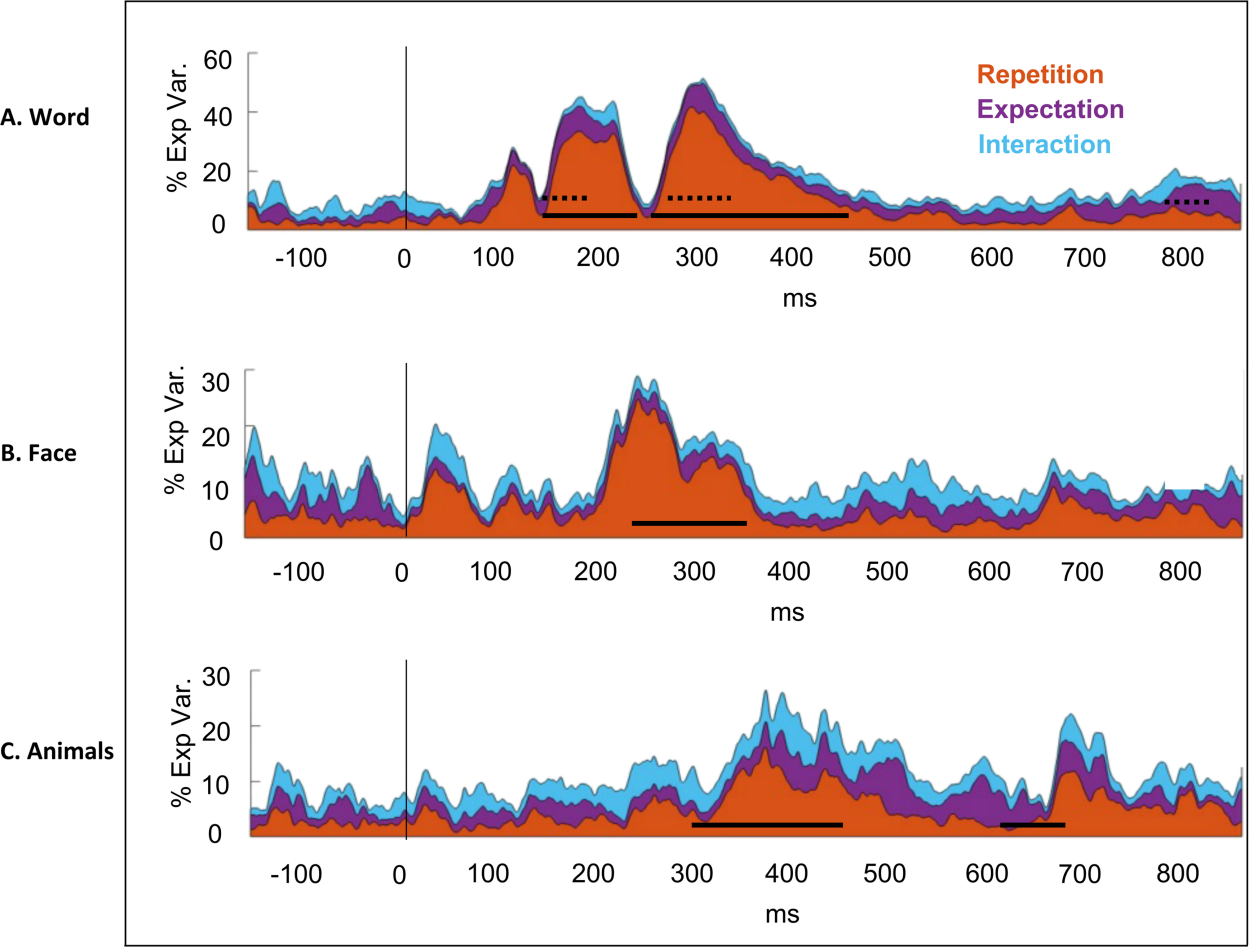
Explained variance and TANOVA results for repetition, expectation and interaction. Panels (A)–(C) display the cumulative percentage of explained variance (y‐axis) over time (x‐axis), and the explained variance for each effect separately (orange = repetition effects, purple = expectation effects, blue = repetition × expectation interaction) for (A) Chinese words, (B) faces and (C) animals. The solid line indicates significant time windows for Repetition effects after the global duration statistics (GDS), while the dotted line indicates significant time windows for Expectation effects. Chinese words (Panel A) show significant main effects of repetition from 151 to 245 ms (partial *η* ^2^ = 0.25) and from 260 to 455 ms (partial *η* ^2^ = 0.21); and significant effect of expectation in three intervals: 148–204 ms (partial *η* ^2^ = 0.08), 294–341 ms (partial *η* ^2^ = 0.08) and 783–847 ms (partial *η* ^2^ = 0.09). Faces (Panel B) show significant main effect of repetition between 209 and 356 ms (partial *η* ^2^ = 0.15). Animals (Panel C) display repetition effect from 327 to 486 ms (partial *η* ^2^ = 0.15) and from 659 to 726 ms (partial *η* ^2^ = 0.08).

As shown in Figure 2A, TANOVA of ERPs to written words revealed significant main effects of repetition in 151–245 and 260–455 ms.^3^ These intervals remained significant after correcting for multiple comparisons by the GDS method. Grand average ERP waveforms elicited by the repeated and alternating conditions at example electrodes are displayed in Figure 5A.

**FIGURE 3:**
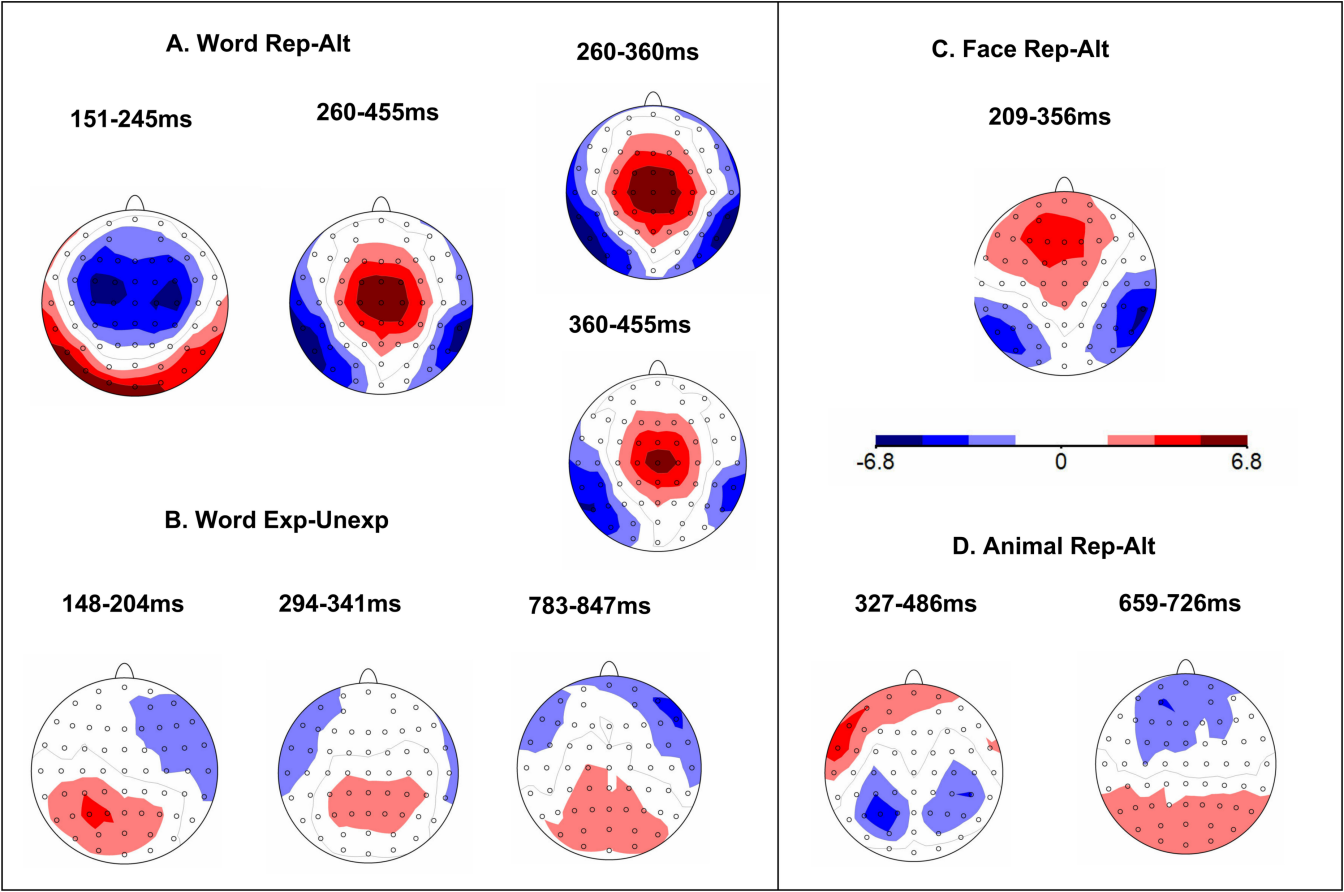
Statistical *t*‐maps for repetition effects for visual words (A), expectation effect (B), repetition effects for faces (C) and animals (D). Alt = alteration, Exp = expectation, Rep = repetition, Unexp = unexpectation. The scale of the t‐map is consistent across three conditions from −6.8 to 6.8.

Significant repetition effects were first observed during a time window of 151–245 ms (partial *η* ^2^ = 0.25, *d* = 1.15), where repeated words elicited more positive ERP waveforms at bilateral occipitotemporal electrodes and more negative (less positive) waveforms at central electrodes compared to alternating words. In the time window 260–455 ms (partial *η* ^2^ = 0.21, *d* = 1.03), repeated words elicited less positive waveforms over bilateral posterior channels and less negative waveforms over frontal and central channels (Figure 3A).

As shown in Figure 2B, for faces, TANOVA revealed a significant main effect of repetition between 209 and 356 ms (partial *η* ^2^ = 0.15, *d* = 0.84). This effect was characterized by more negative‐going (or less positive) waveforms at bilateral occipitotemporal electrodes and more positive (less negative) waveforms at frontal and central channels for repeated faces (Figure 3C).

**FIGURE 4:**
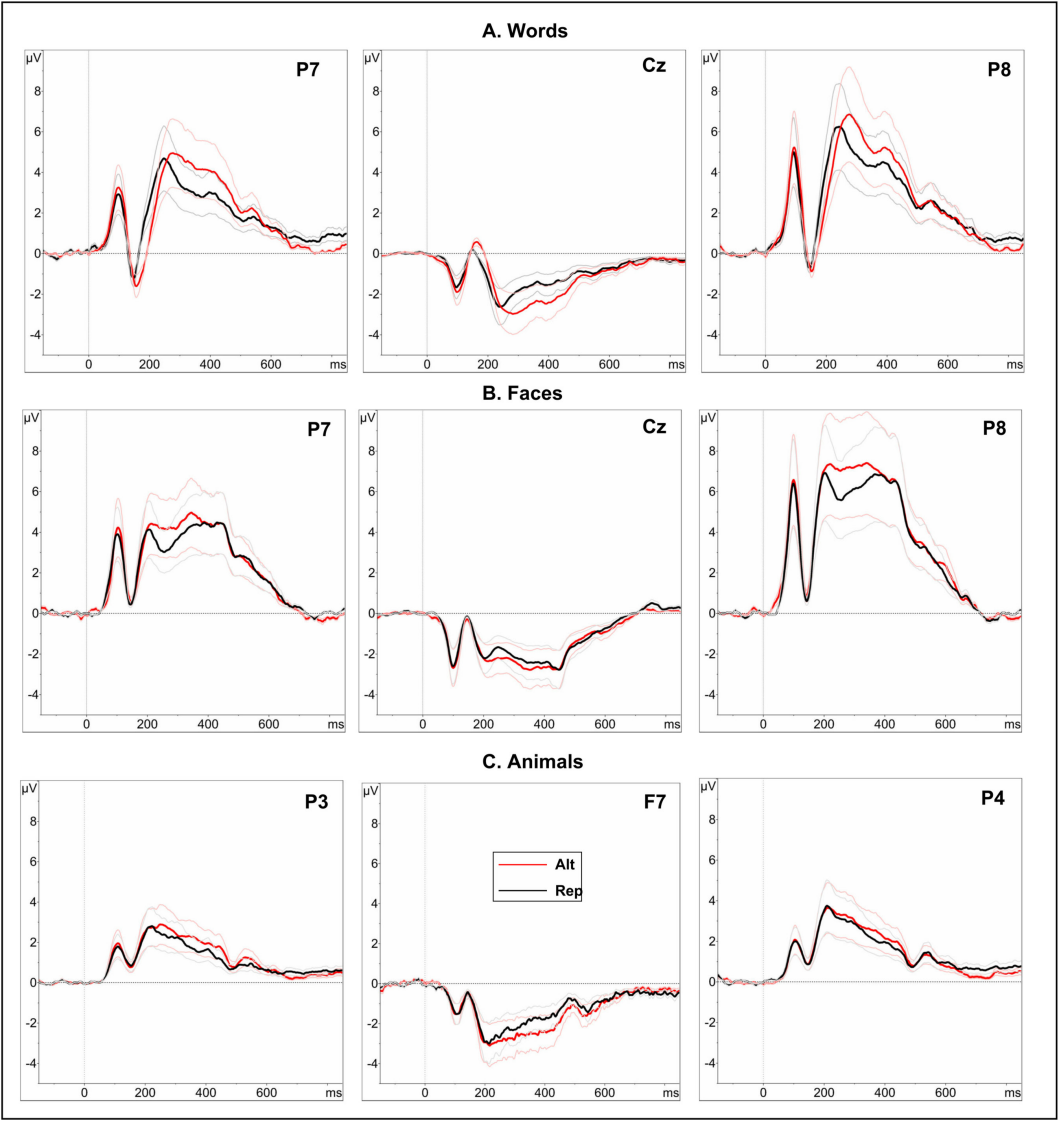
Grand‐averaged ERP waveforms at exemplary electrodes illustrating repeated and alternating conditions (A: words, B: faces, C: animals) irrespective of expectation with standard error traces in the ERPs. Alt = alteration, Rep = repetition.

As shown in Figure 2C, for animals, TANOVA revealed a significant main effect of repetition between 327 and 486 ms (partial *η* ^2^ = 0.15, *d* = 0.84). This effect was significant after correcting for multiple comparisons and characterized by more negative‐going (less positive) waveforms at bilateral parietal electrodes and more positive‐going (less negative) waveforms at left frontal channels for repeated animal pictures (Figure 3D). TANOVA also revealed a significant main effect of repetition between 659 and 726 ms over frontal and posterior channels (partial *η* ^2^ = 0.08, *d* = 0.59). Grand average ERP waveforms evoked by repeated and alternating conditions for faces and animals, respectively, are displayed for example electrodes in Figure 4B,C.

#### 3.2.2 Expectation Effects

For visual words, TANOVA revealed main effects of expectation during three time windows: 148–204 ms (partial *η* ^2^ = 0.08, *d* = 0.59), 294–341 ms (partial *η* ^2^ = 0.08, *d* = 0.59) and 783–847 ms (partial *η* ^2^ = 0.09, *d* = 0.63). The late time window (783–847 ms) showed significant effects over right frontal and posterior electrodes (Figure 2B). However, the repetition effects were consistent across both expected and unexpected words throughout the epoch, with no significant interaction clusters observed. Moreover, neither the main effects of expectation nor the interactions Repetition and Expectation reached statistical significance at any time point for faces and animals.

#### 3.2.3 Shared and Distinct Repetition Effects Across Stimulus Types

Despite some variance in timing and scalp distributions, we identified a common repetition effect (N250r) across faces, animals and visual words (Figure 3). The N250r effect for visual words (260–455 ms), with more negativity for repeated words at inferior temporal electrodes, was particularly over the left hemisphere, and more positivity in the central electrodes. For faces, repetition effects were observed within 209–356 ms, with more negativity for repeated faces at inferior temporal electrodes, particularly over the right hemisphere. A similar N250r‐like effect was identified for repetitions of pictures of animals during the 327–486 ms time window over frontal and bilateral parietal electrodes. Moreover, pairwise comparisons with normalized amplitudes were conducted to determine if N250r effects for words, faces and animals were distinct. The results revealed significantly different topographical distributions between faces and animals (*p* = 0.001, partial *η* ^2^ = 0.13, *d* = 0.77), between words and animals (*p* =《》0.0002, partial *η* ^2^ = 0.29, *d* = 1.28) and between words and faces (*p* = 0.005, partial *η* ^2^ = 0.11, *d* = 0.70). These findings suggest that the N250r effects exhibit unique topographical distributions for faces, animals and visual words.

**FIGURE 5:**
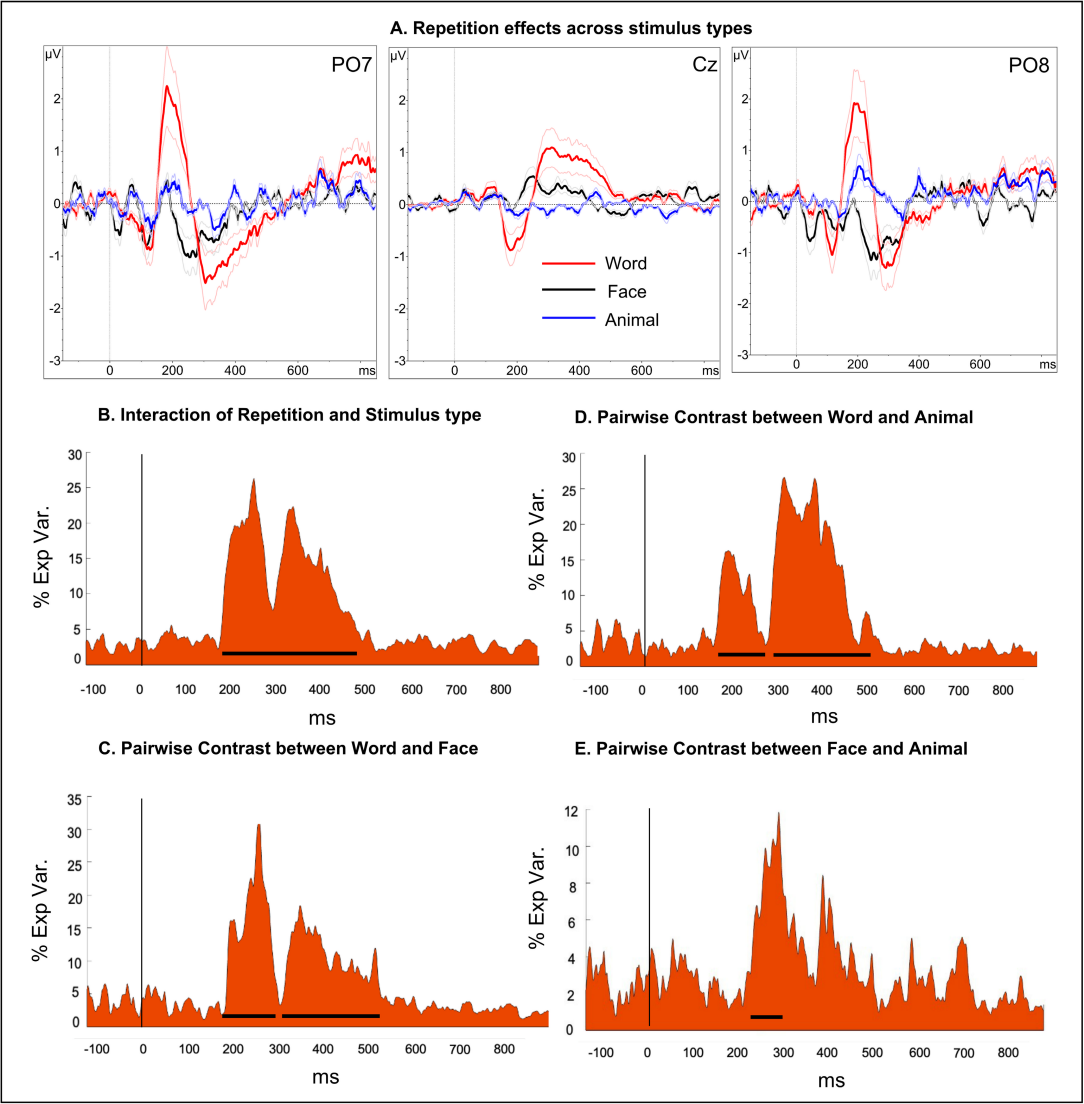
Repetition effects across three stimulus types. (A) Grand‐averaged ERPs at electrodes PO7, Cz and PO8 for words (red), faces (black) and animals (blue). Shaded regions indicate the standard error of the mean. (B) The cumulative percentage of explained variance (partial *η* ^2^) (y‐axis) for the interaction of repetition (repeated vs. alternated) and stimulus type over time (x‐axis). The solid black line indicates significant time windows after multiple‐comparison correction (150–442 ms). (C–E) Time course of the partial *η* ^2^ for pairwise contrasts (C) between word and face, (D) between word and animal and (E) between face and animal. Repetition effects for written words differed significantly from faces at 152–259 ms (partial *η* ^2^ = 0.18) and 277–481 ms (partial *η* ^2^ = 0.11), and from animals at 151–235 ms (partial *η* ^2^ = 0.13) and 265–441 ms (partial *η* ^2^ = 0.19). The repetition effects for faces and animals differed significantly at 233–289 ms (partial *η* ^2^ = 0.91).

To investigate temporal differences in repetition effects across stimulus types, we conducted TANOVAs with non‐normalized amplitudes with factors repetition (Repeated vs. Alternated) and stimulus types (word, face and animals). The results showed a significant interaction of these factors between 150 and 442 ms (Figure 5B). Pairwise contrasts between the stimulus conditions with non‐normalized amplitudes revealed that repetition effects for written words were significantly different compared to faces at 152–259 ms (*p* < 0.05, partial *η* ^2^ = 0.18, *d* = 0.94) and 277–481 ms (*p* < 0.05, partial *η* ^2^ = 0.11, *d* = 0.70), and as compared to animals at 151–235 ms (*p* < 0.05, partial *η* ^2^ = 0.13, *d* = 0.77) and 265–441 ms (*p* < 0.05, partial *η* ^2^ = 0.19, *d* = 0.97). Moreover, the repetition effects between faces and animals differed significantly at 233–289 ms (*p* < 0.05, partial *η* ^2^ = 0.09, *d* = 0.63) (Figure 5B–E). These results suggest that repetition effects for written words occurred earlier than for faces and animals, and repetition effects for faces occurred earlier than for animals.

Given the relatively broad time window of the N250r effect for visual words (260–455 ms) and the absence of any N400 effect, as part of an exploratory analysis, we divided this time window into two segments (260–360 ms, 360–455 ms, see Figure 3A). To examine the temporal dynamics of potential N250‐to‐N400 transitional effects, we conducted topographical analyses between early (260–360 ms) and late (360–455 ms) intervals using the normalization procedure, which is done by dividing by GFP (McCarthy and Wood 1985). Results revealed significant topographical dissociations between intervals (*p* = 0.002, *η* ^2^ = 0.12, *d* = 0.74), indicating distinct spatial configurations of repetition effects across these time windows.

## 4 Discussion

This study examined category‐specific P(rep) effects in ERPs using a cued repetition probability paradigm that orthogonally manipulated stimulus repetition and expectation across three categories: visual words, faces and animals. Additionally, we investigated shared and distinct neural mechanisms of repetition effects across these categories within a single experimental context. Results revealed temporally distinct repetition effects for words (151–245, 260–455 ms) with expectation effects emerging during later processing stages (148–204, 294–341, 783–847 ms). However, we failed to find EEG evidence for P(rep) effects during word processing. Face stimuli elicited N250r effects (209–356 ms), while animals showed analogous effects within a later window (327–486 ms). Neither category demonstrated a modulation of the repetition effect by repetition probability or expectancy.

### 4.1 Stimulus Repetition Effects

We first examined repetition effects in the three stimulus types, specifically faces, words and animals. TANOVA results demonstrated that repetition effects were evident across all stimulus types, albeit with differences in timing and scalp topography. The following sections first discuss the common N250r effects observed across all categories, followed by an examination of category‐specific repetition effects, with particular emphasis on the distinctions between visual words and faces relative to animal pictures.

The N250r component, characterized by enhanced right–lateralized negativity at occipitotemporal electrodes (200–300 ms), is associated with face repetition, particularly for identical images of familiar faces (Schweinberger and Neumann 2016). Our findings revealed significant face repetition effects within 209–356 ms poststimulus onset, with a characteristic right‐hemispheric occipitotemporal distribution, consistent with the canonical N250r. While initially identified in face processing, the N250r extends beyond face specificity, emerging across multiple stimulus categories (Engst et al. 2006; Martín‐Loeches et al. 2005). In our study, visual words elicited N250r effects within 260–455 ms, showing increased left‐lateralized occipitotemporal negativity, aligning with previous findings for written names. Thus, Pickering and Schweinberger (2003) found an increased left temporal negativity (N250r during 220–300 ms for repeated written familiar names). Animal stimuli produced N250r‐like effects during 327–486 ms, characterized by frontal and bilateral parietal distributions, corroborating prior research on common objects (Martín‐Loeches et al. 2005; Engst et al. 2006), which reported the N250r effect for repetitions of common objects (Martín‐Loeches et al. 2005) and familiar buildings (Engst et al. 2006). The N250r has been interpreted as indicating the retrieval of structural knowledge from long‐term memory (Bindemann et al. 2008; Wiese et al. 2013; Zimmermann and Eimer 2013). Therefore, these shared effects suggest a domain‐general mechanism within the visual modality for accessing and comparing stored structural representations within different visual stimulus categories. While RS and priming effects are observed in non‐visual modalities (e.g., see Grill‐Spector et al. 2006 for review), the commonalities reported for the N250r in this study appear to be specific to the visual domain, reflecting modality‐specific processes for structural representation retrieval.

We also found that the topographical distribution of N250r effects for words, faces and animals was significantly different. There was a trend for face and visual words to exhibit different topographies of N250r, with maxima at temporo‐occipital negativity (PO7, PO8) and anterior positivity. In contrast, animals showed somewhat more centred but still bilateral parietal negativities and a prefrontal positivity. These topographical variations might reflect underlying differences in how structural representations for faces, words and animals are stored and retrieved from memory. The structural information for objects might be retrieved from different cortical areas compared to face and visual words.

There is also an issue regarding the presence of N250r for unfamiliar faces and animals in this study. While the N250r is typically more pronounced for familiar faces (Schweinberger and Neumann 2016), evidence indicates that an amplitude increases with face familiarization, reflecting the acquisition of structural codes during learning (Zimmermann and Eimer 2013). Previous research suggests that the N250 itself increases when unfamiliar faces are presented repeatedly (Sommer et al. 2021; Tanaka et al. 2006) and posited that N250 indexes both pre‐experimentally familiar and newly acquired face representations formed during the experiment. Therefore, it has been proposed that the N250 is associated with the processing of familiar faces at the subordinate representational level (Tanaka et al. 2006; Wiese et al. 2019). In the present study, despite initial stimulus unfamiliarity, each item's fourfold presentation may have facilitated the development of structural representations through rapid perceptual learning, enabling subsequent retrieval processes typically associated with familiar stimuli.

The present study also identified distinct repetition effects for visual words and faces compared to animals. Notably, the repetition effects emerged earlier for visual words than for faces and animals, with the N250r effect for visual words preceded by an earlier effect within 151–245 ms, occurring during the N1‐P2 transition. This suggests latency shifts and accelerated temporal dynamics of the N1 component, indicating the facilitated processing of repeated words. Prior research has shown that familiarity can modulate repetition effects for both faces and visual words (Guillaume et al. 2009; Maurer et al. 2023; Nie et al. 2016). For example, Maurer et al. (2023) reported that the repetition effect in the N1 offset was significantly larger for familiar Chinese characters compared to unfamiliar Korean characters.

The temporal advantage for words can be attributed to both familiarity effects and inherent stimulus properties. This study used high‐frequency word stimuli to which participants had considerable prior exposure and thus higher familiarity relative to faces and animals. This difference likely contributed to more efficient processing associated with word repetition. It is important to note that familiarity refers to individual stimuli, whereas expertise refers to extensive experience with a stimulus category, that is, participants may have expertise with both words and faces due to their frequent everyday encounters with these categories. However, their familiarity was specifically with the visual words, not with the particular faces or animal images presented during the experiment.

In addition, it is likely that stimulus properties themselves, such as high spatial frequency, influence early visual processing, for example, written words are complex visual stimuli containing high spatial frequencies due to the fine detail required to discern letters and words (Jordan et al. 2017), which may lead to the early repetition effects observed in the ERPs.

Second, the N400‐like repetition effect was observed with visual words, but not with faces and animals. The N400 effect, characterized by a decreased centro‐parietal negativity, indicates the facilitation of semantic processing of repeated words, consistent with previous research (Bentin et al. 1985; Rugg 1985, 1990; Van Petten et al. 1991). The 360–455 ms window aligns with documented N400 latencies, potentially overlapping with LPC/P600 effects related to memory encoding and retrieval (Olichney et al. 2000; Paller and Kutas 1992; Van Petten et al. 1991). The reduced N400 may indicate facilitated semantic processing following the repeated word presentation (Bentin et al. 1985; Van Petten et al. 1991; Rugg 1985, 1990). The N400 repetition effect was observed for visual words but not for faces and animals, likely due to local stimulus familiarity developed through repeated exposure in the experiment. It is conceivable that participants may gradually acquire perceptual familiarity with faces and animals, facilitating structural representations; however, retrieving individual‐level semantic information for these novel stimuli from long‐term memory poses challenges. In contrast, the high‐frequency words used in this study are associated with deeper semantic knowledge, facilitating the retrieval of specific individual‐level semantic information. However, caution is warranted in interpreting these findings, especially given the exploratory nature of segmenting ERP responses into the 260–360 ms and 360–455 ms windows.

### 4.2 Stimulus Expectation Effects

Interestingly, we found effects of expectation during different time windows on visual words. In the early window (148–204 ms), we found a reduced negativity at midposterior electrodes and increased positivity over left frontal channels for expected stimuli, consistent with early expectation influences on N1 offset as reported in prior studies (Todorovic and de Lange 2012; F. Wang and Maurer 2020). Notably, F. Wang and Maurer (2020) found that category‐level expectations enhance visual‐orthographic processing within 200 ms, highlighting the facilitative role of early expectation in word recognition. During a later window (294–341 ms), expectation effects manifested significantly over right frontal and posterior electrodes, consistent with the topography and latency of stimulus expectation effects in similar repetition probability designs manipulating expectations (Feuerriegel et al. 2018; Summerfield et al. 2011). The overlapping latencies of these early expectation effects with the N170 and N250 repetition effects suggest additive rather than interactive influences, supporting the two‐stage model of independent repetition and expectation processes proposed by Grotheer and Kovács (2016). Finally, in the late time window spanning 783–847 ms, we identified expectation effects over the posterior electrodes and right frontal channels. The topography of these late expectation effects differed from the earlier effects, suggesting that early and late ERP components may index qualitatively different aspects of expectation. This distinction provides insights into the complex nature of expectation effects, suggesting that expectation effects captured in fMRI studies may reflect a mixture of these temporally distinct effects, emphasizing the temporal aspects of expectation dynamics.

### 4.3 Repetition Probability Effects

The second important finding of the current study is that, whereas we observed repetition effects across faces, words and animals, there was no significant modulation by repetition probability for any of the three stimulus categories. This lack of modulation aligns with previous findings where expectation did not influence RS (Feuerriegel et al. 2018; Kaliukhovich and Vogels 2011; Todorovic and de Lange 2012; Vinken et al. 2018). For instance, Todorovic and de Lange (2012) found that repetition and expectation effects operate on distinct time segments in the MEG with pure tone stimuli. Similarly, in ERPs, unique spatiotemporal patterns of repetition and expectation were identified for faces (Feuerriegel et al. 2018), and an additive effect of repetition and expectation was found in fMRI (Grotheer and Kovács 2015; Kovács et al. 2013; Vinken et al. 2018). These results do not support a general PC account of RS, which posits that perceptual expectations modulate repetition effects (e.g., Auksztulewicz and Friston 2016) and reduced prediction error signalling when expectations are met (Clark 2013; Friston 2005; Summerfield and de Lange 2014). To the contrary, our findings contribute to a growing body of evidence supporting the two‐stage model, suggesting that the neural mechanisms underlying expectation and repetition effects are distinct and separable (Grotheer and Kovács 2016).

Although the theoretical predictions regarding expectancy effects are not fully supported by the empirical data, we believe that the PC model and the two‐stage model are not inherently incompatible. Instead, they may represent distinct mechanisms operating within a broader predictive framework. The predictive processes may arise in multiple, hierarchically organized stages. Findings by Wacongne et al. (2011) from an auditory novelty paradigm support the idea that predictive processes may operate in multiple hierarchically organized stages, ranging from lower level predictions based on local transitions to a higher level expectation processes based on the overall stimulus pattern. Therefore, in the current study, the lack of modulation of repetition effects by repetition probability may suggest that predictions arise from stimulus repetition and expectations due to the knowledge of stimulus probability from distinct processing levels. In other words, the level of representation required for repetition predictions might be lower in this hierarchy than the level of representation required for longer range expectations of repetition probabilities.

Several alternative explanations can be offered to explain the absence of expectancy modulation of RS (P(rep) effects). One possibility is that participants did not learn the association between the subcategories of the first stimulus (S1) and the repetition probability of the second stimulus (S2), preventing the formation of perceptual expectations. However, this seems unlikely as participants were explicitly instructed to memorize the association between the S1 identity and the repetition probability before the experiment. Furthermore, based on self‐reports collected after the EEG experiment, most participants were aware of the contingencies between the identity of S1 and the repetition probabilities of S2.

Moreover, while participants were explicitly informed about repetition probabilities, our findings revealed no significant effects of these probabilities on neural responses. This may suggest a potential contribution of attentional and executive processes in the cuing probability design. It appears that even when participants are aware of the associations, their brains may not necessarily use this information for making predictions. This explanation is supported by various previous findings. For instance, Summerfield et al. (2008) demonstrated implicit learning of repetition probabilities without explicit instructions, whereas other studies, such as Todorovic and de Lange (2012), reported that explicit awareness of cued probabilities had independent and additive effects on neural responses. The apparent inconsistency across studies suggests that explicit and implicit expectations might not tap into the same underlying neuronal mechanisms. Future studies should distinctly assess implicit and explicit expectations using different experimental paradigms to clarify whether these two forms of expectations engage similar or distinct neural processes. Second, the repetition probability effect might be stimulus category‐specific and dependent on prior experiences (visual expertise hypothesis, Grotheer and Kovács 2014). Indeed, previous research has demonstrated P(rep) effects for faces (Kovács et al. 2013; Larsson and Smith 2012) and familiar alphabetic letters (Grotheer and Kovács 2014). However, the present study did not find evidence of significant repetition probability effects for any of the three categories, even for written words and faces, which are typically associated with high visual expertise. The consistent absence of P(rep) effects across three different stimulus types may suggest that expectation modulations of repetition may not be inherently related to visual expertise. In this sense, the null findings of this study are crucial as they offer novel insights into the relations of P(rep) effects and visual expertise. Third, while some argue that expectation effects on RS may be specific to BOLD signals rather than EEG measures with visual stimuli (Den Ouden et al. 2023), recent evidence challenges this view. EEG studies have demonstrated expectation‐modulated RS after 300 ms in visual word recognition (Song et al. 2024) and faces (Summerfield et al. 2011), and between 100 and 200 ms for pure tones (Todorovic and de Lange 2012). For instance, our recent study examining P(rep) effects during visual word recognition using Block RS design found that expectations modulate RS at late stages (after 300 ms) but not at early stages (Song et al. 2024). This evidence may suggest that probability‐based top‐down influences on RS in EEG measures.

Finally, the absence of P(rep) effects in our study may also reflect methodological differences in expectation manipulation. Studies reporting repetition probability effects typically employ block designs, where repetition frequency varies between blocks (Grotheer and Kovács 2014; Kovács et al. 2013; Larsson and Smith 2012; Song et al. 2024; Summerfield et al. 2011). In contrast, event‐related designs, which link current stimulus properties to upcoming repetition probabilities, often show independent repetition and expectation effects (Den Ouden et al. 2023; Feuerriegel et al. 2018; Grotheer and Kovács 2015). Our failure to find P(rep) effects using an event‐related design, despite explicit probability instructions, aligns with other recent findings (Den Ouden et al. 2023) and suggests that event‐related expectation manipulations may be insufficient to modulate RS. Future research should systematically investigate how different experimental approaches impact expectation‐based RS modulation.

As a final point, the interpretation of ERP amplitude modulations as evidence for or against PC remains debatable. Our study, while not providing support for the general PC account of RS, does not refute the PC theory. The current study may only provide evidence for the failure of using of P(rep) as a general indicator of PC in the cueing repetition probability design. As Bowman et al. (2023) note, failure to falsify neither implies unfalsifiability nor does falsifiability imply falsification. Future investigations employing intracranial EEG, high‐resolution fMRI or advanced deep learning approaches may provide more definitive tests of PC theories.

### 4.4 Limitations

We acknowledge some limitations in the current study. First, we used the repetition probability paradigm adopted from Grotheer and Kovács (2015) in which participants were instructed to identify properties of the first stimulus (S1), such as the sex of faces. However, this design might shift participants' focus towards the identity of S1, away from the repetition probability, despite explicit instructions to focus on the latter. This potential diversion in attention could explain the absence of significant expectation effects or interactions driven by repetition. Future studies might benefit from using tasks that are more directly related to repetition probability. This design might increase top‐down expectation effects, making them more likely to modulate the EEG responses associated with RS. Second, while we examined stimuli of different visual expertise (faces, words and animals), the categories used inherently also differ in other properties, such as semantic depth and low‐level visual features. Future research should investigate P(rep) effects within a given category but across familiarity levels (e.g., Abdel Rahman 2011; Abdel Rahman and Sommer 2008) to better isolate familiarity effects while controlling for confounding stimulus properties. Third, while effect sizes were very large for main effects of repetition in the current and in previous studies (with Cohen's *d* ranging from 0.9 to 2.6 depending on the design and measure), the effect size for an interaction with expectation may be considerably smaller. In a recent study using TANOVA with a block design, the effect size for the expectation‐by‐repetition interaction was *d* = 0.58 (Song et al. 2024). Therefore, the current study may have had only borderline power to detect an interaction, and larger samples and/or more specific measures may be needed in future studies to detect such interactions. An additional potential limitation of our study is the initial neglecting in power calculations regarding the shape of interaction effects. As Sommet et al. (2023) note, the shape of an interaction can significantly influence both effect size and required statistical power. Variations in interaction patterns—ranging from simple crossovers to more complex forms—can affect power calculations. By not accounting for these factors, our study may have underestimated the required sample size, potentially compromising the reliability of our findings. Future research should incorporate these considerations into power analyses to ensure accurate detection of interactions. Finally, we acknowledge that our analyses were confined to group‐level mean differences, and we recognize that this approach may overlook individual‐level effects or variations in other aspects of the distribution (Rousselet et al. 2016, 2017).

## 5 Conclusions

In conclusion, our study revealed both shared and distinct repetition effect mechanisms across Chinese visual words, faces and animals. Temporal distinctions emerged, with repetition effects manifesting earliest for words, followed by faces, then animals. While an N250r effect was present across all categories, its timing and distribution varied, suggesting familiarity‐dependent modulation. Notably, despite explicit repetition contingencies and the use of highly familiar stimuli like visual words, expectation did not modulate repetition effects in any stimulus category, indicating that stimulus‐based repetition expectations alone may be insufficient to modulate repetition effects. Thus, the present findings fail to support a PC account of RS effects for visual categories in which repetition effects are modulated by expectation.

## Author Contributions

**Bingbing Song:** conceptualization, data curation, formal analysis, investigation, methodology, software, visualization, writing – original draft. **Werner Sommer:** conceptualization, methodology, supervision, writing – review and editing. **Urs Maurer:** conceptualization, formal analysis, funding acquisition, methodology, software, supervision, writing – review and editing.

## Conflicts of Interest

The authors declare no conflicts of interest.

### Peer Review

The peer review history for this article is available at https://www.webofscience.com/api/gateway/wos/peer‐review/10.1111/ejn.70047.

## Supporting information


**Figure S1.** Distribution of linguistic properties across animate and inanimate nouns. (A) Word frequency (log‐transformed counts per million). (B) Total stroke counts. (C) Repetition rates (times per trial). Error bars represent standard deviations. Animate and inanimate nouns are differentiated by colour (animate: blue; inanimate: orange). 227 × 240mm (300 × 300 DPI).

**Figure S2.** Scatter plots display mean accuracy and mean reaction time for different conditions: words, faces and animals. Conditions: Rep_Exp (repetition‐expected), Rep_Unexp (repetition‐unexpected), Alt_Exp (alternation‐expected) and Alt_Unexp (alternation‐unexpected).

## Data Availability

The data are available at https://osf.io/xd92h/.

## Endnotes

^1^
This experiment involved 31 Hong Kong‐born participants (22 females, mean age = 19.32, SD = 1.78). They were tasked with categorizing images of faces, words and objects by identity, with accuracy measured across the different stimulus types.

^2^
In Chinese writing system, a word is often composed of two characters, and sometimes, one character may appear in both positions of the two‐character word. The character repetition times refer to the frequency with which characters were repeated within the two‐character words.

^3^
It is important to note that the GDS, as a cluster‐based method, provides a single *p* value at the cluster level and therefore does not allow for precise inferences about the exact onset or offset of the observed effects (Rousselet 2025; Sassenhagen and Draschkow 2019).
