# Supplementary figures and images for: Discrete Repetition Effects for Visual Words Compared to Faces and Animals, but No Modulation by Expectation: An Event‐Related Potential Study
Source: Eur J Neurosci. 2025 Mar 3;61(5):e70047. doi: 10.1111/ejn.70047 (PMC11876721; doi:10.1111/ejn.70047)

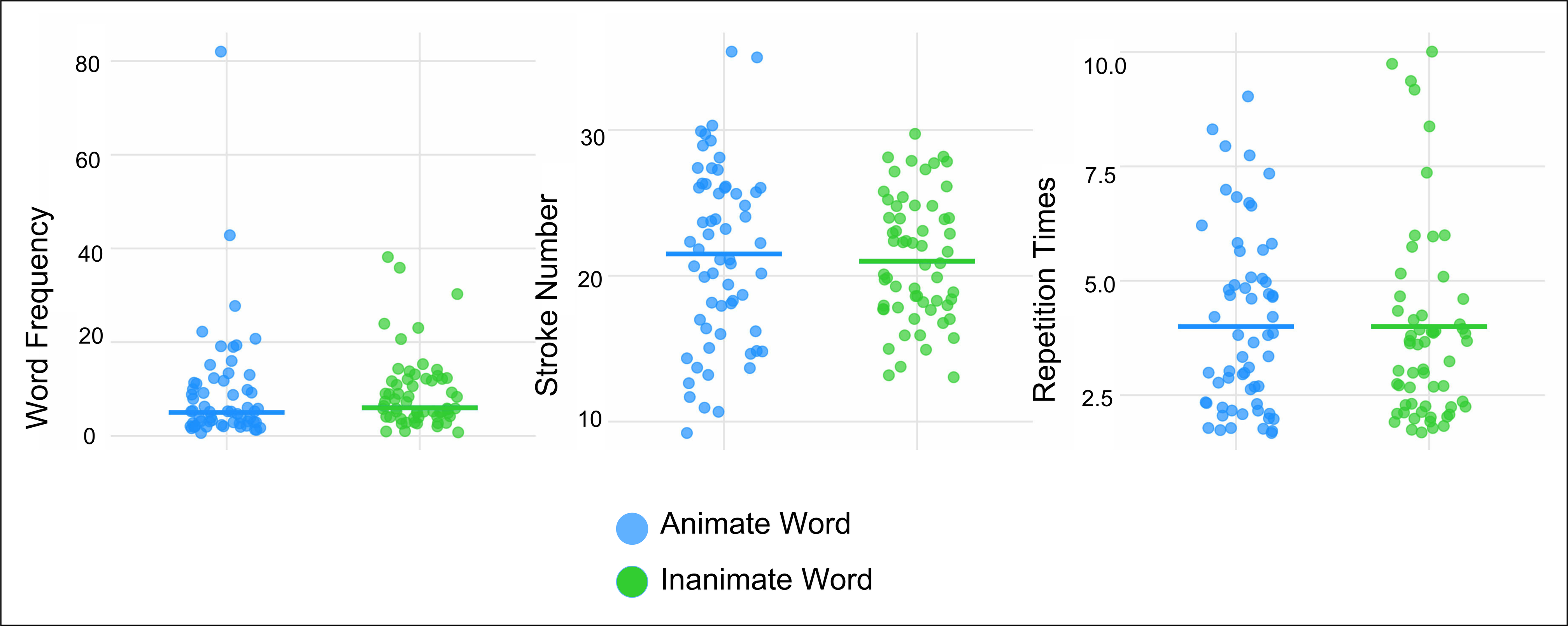

Supplement: Supplementary file 1 — Figure S1. Distribution of linguistic properties across animate and inanimate nouns. (A) Word frequency (log‐transformed counts per million). (B) Total stroke counts. (C) Repetition rates (times per trial). Error bars represent standard deviations. Animate and inanimate nouns are differentiated by colour (animate: blue; inanimate: orange). 227 × 240mm (300 × 300 DPI). [file EJN-61-0-s001.tif]

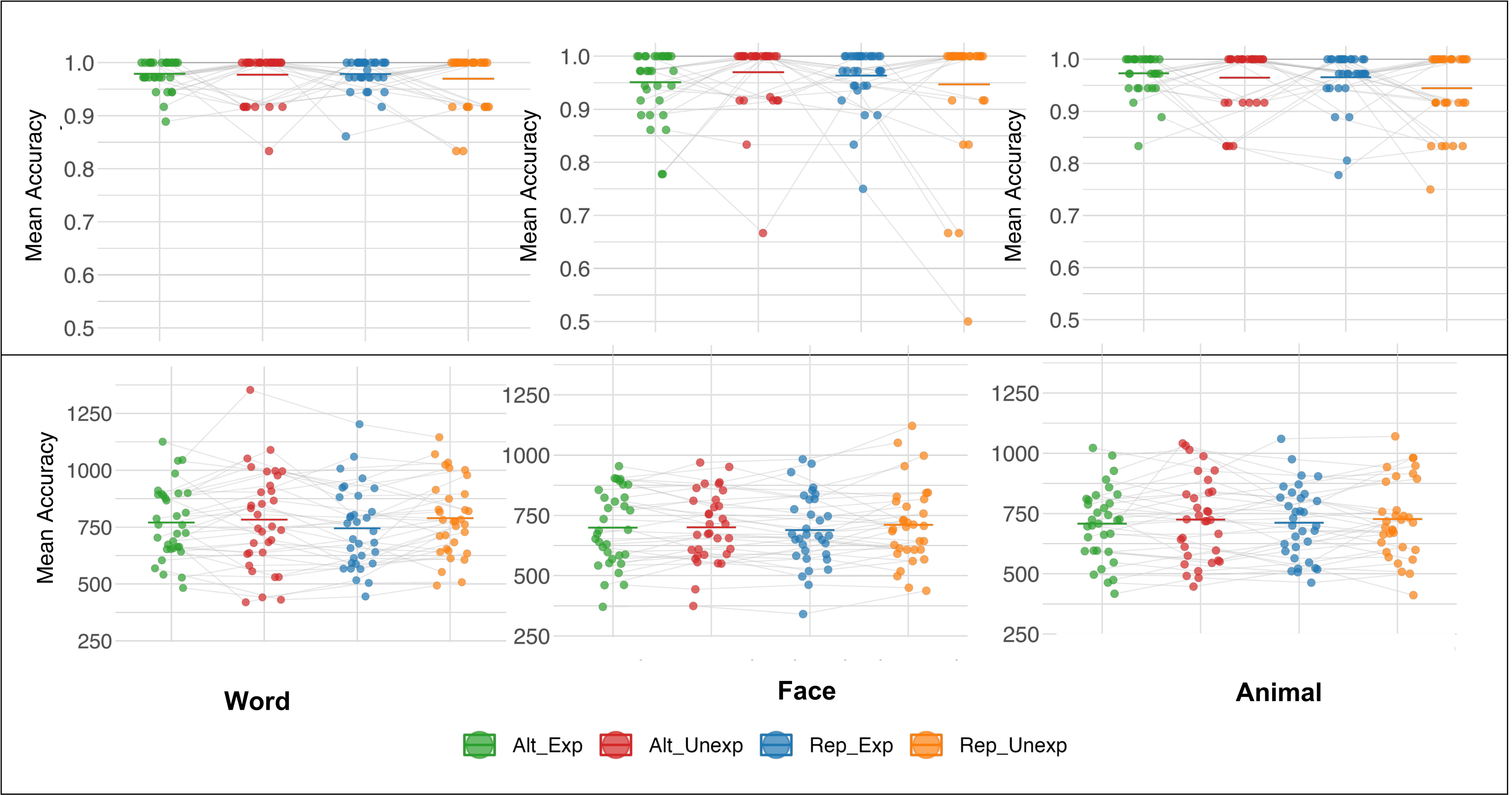

Supplement: Supplementary file 2 — Figure S2. Scatter plots display mean accuracy and mean reaction time for different conditions: words, faces and animals. Conditions: Rep_Exp (repetition‐expected), Rep_Unexp (repetition‐unexpected), Alt_Exp (alternation‐expected) and Alt_Unexp (alternation‐unexpected). [file EJN-61-0-s002.tif]
